# Leisure Satisfaction, Personality, and Psychosexual Adjustment Among College Students: A Latent Profile Analysis

**DOI:** 10.3389/fpsyg.2022.895411

**Published:** 2022-05-19

**Authors:** Ximei Xia, Xiaotian Wang, Yuting Wang

**Affiliations:** Department of Psychology, Qingdao University, Qingdao, China

**Keywords:** personality, psychosexual adjustment, leisure satisfaction, latent profile analysis, college students

## Abstract

Although the factors influencing sexual health have been explored by researchers, the impact of leisure and personality on psychosexual adjustment and the interaction of these two factors remain unknown. This study investigated the relationship between leisure satisfaction and psychosexual adjustment based on the compensation theory and the social learning theory. The differences in psychosexual adjustment across different personality types were also explored. Finally, we examined the interaction between personality and leisure satisfaction based on the personal-environment fit models. The participants in this study were 1,161 college students. The results supported all the hypotheses proposed. There was a significant positive correlation between leisure satisfaction and psychosexual adjustment. Participants of four personality types (the overcontrolled, high-moderate, low-moderate, and resilient groups) had different performance in psychosexual adjustment. The resilient group had the highest scores, while the overcontrolled group had the lowest scores. The results suggest that there is an interaction between personality and leisure satisfaction. Our research could enrich the research contents of leisure and personality and provide a practical basis for the improvement of college students in psychosexual adjustment.

## Introduction

Psychosexual adjustment is a psychological variables that affects sexual functioning, including thoughts, emotions, behaviors, and interpersonal relationships (Sprankle, 2015). When researchers investigate sexual functioning and sexual health, they mostly discuss them in terms of physiological aspects (Cherven et al., 2021). However, adolescents and young adults are accompanied by a series of psychosocial developments as well. In China, young people tend to be more open-minded about sexuality along with social progress (Song, 2015). However, current inappropriate sex education runs counter to the trend toward openness among the youth. Consequently, many teenagers still have rigid attitudes about gender and sexuality as well as misconceptions about puberty and masturbation (Sa et al., 2021). This probably causes adolescents to have difficulty in adjusting. Especially after they enter college, as their scope of interpersonal communication expands, they may be confronted with the challenges of forming intimate relationships in pre-adulthood (Erikson, 1963), including issues related to psychosexual adjustment. In light of this situation, our study mainly focused on the psychosexual adjustment of college students.

In general, psychosexual adjustment can be divided into three domains: psychosexual selfhood (intrapersonal functioning such as body image and self-perception), psychosexual socialization (related to the interaction of the individuals with their social environment), and sexual/intimate behavior (sexualized behaviors and experiences; Dekker et al., 2017). Scholars also have measured psychosexual adjustment from 12 dimensions, including internal sexual control (individuals feel their sexual life under their control; Alley and Strassberg, 2020).

Previous studies have found that psychosexual adjustment is linked to stress (Arya et al., 2019), cancer and treatment (Barnett et al., 2016; Rizzuto et al., 2021), puberty development (Dwyer, 2020), leisure (Williams et al., 2020), and personality (Thompson et al., 2019; Hashemi, 2020), among which leisure is of great significance to everyone, especially college students who have plenty of free time (Das and Barman, 2019). In addition, the role of individual personality on sexual psychology has also been of interest (Frías et al., 2017). As a result, our study focused on the relationship among leisure satisfaction, psychosexual adjustment, and personality.

### Leisure Satisfaction and Psychosexual Adjustment

Leisure satisfaction refers to positive views or feelings obtained in leisure activities, which reflects the overall satisfaction degree of leisure experience (Beard and Ragheb, 1980). According to the compensation theory (Wilson, 1980), individuals can compensate for negative aspects in other areas of life through well-experienced leisure activities. Some hold that leisure satisfaction may even be more important than other sources of life satisfaction (Tokay Argan and Mersin, 2021). From an ecological perspective, both positive leisure activities and consequent flow experiences (wonderful experiences of being fully involved in the present moment) not only can help prevent problematic behaviors such as aggression (Park et al., 2017) and substance use (Santini et al., 2020) among young people but also promote their development and adjustment (Mahoney et al., 2009; Freire and Teixeira, 2018). Specifically, excellent leisure experience is associated with a clear sense of self-identity (Mansfield et al., 2020), the formation of social relationships (Kim et al., 2016a), and relief from stress (Chun et al., 2021). All these factors are conducive to the development of psychosexual adjustment (Bigras et al., 2015; Kim and Jang, 2020). Considering leisure is a kind of social construction, Bandura’s social learning theory (Bandura and Walters, 1977) can provide us insights into the understanding of how people promote their psychosexual adjustment through leisure activities. Positive participation and meaningful experience in leisure activities allow the youth to learn how to socialize properly (Granzin and Haggard, 2000) and gain interpersonal support and confidence (Kim et al., 2016b), which have an important impact on their psychosexual adjustment (Parchomiuk, 2019; Barrada and Castro, 2020).

In contrast, a lack of pleasure in leisure seems to be associated with an unhealthy status. For example, leisure boredom could help predict substance use and Internet use in adolescents (Weybright et al., 2015; Wang, 2019). In the field of sexual psychology, studies have shown a correlation between leisure boredom and adolescent risky sexual behaviors (Miller et al., 2014). Based on these facts, we proposed the following first hypothesis:

*H1*: Leisure satisfaction is positively correlated with psychosexual adjustment.

### Personality and Psychosexual Adjustment

Personality is a mode of thinking, feeling and behaving that help to differentiate people from each other. It affects a person’s response to all kinds of situations (Caspi, 1998). Personality is relatively stable throughout life even during adolescence, fluctuating only within a certain range (Brown and Prinstein, 2011). The five-factor model of personality (Van Leeuwen et al., 2004) is one of the most widely used trait models with high reliability and validity at different developmental stages of the life cycle (De Clercq et al., 2009). It measures personality from five dimensions: extraversion, openness, agreeableness, conscientiousness, and neuroticism (also known as emotional stability). However, individuals are usually affected by different trait dimensions simultaneously (Atkins and Hart, 2008). According to the five-factor model, some studies have confirmed that the personality structure can be divided into four classes: the moderate, protected, vulnerable, and undercontrolled groups (De Clercq et al., 2012) or into three classes: the resilient, undercontrolled, and overcontrolled groups (Zhang et al., 2015). In a longitudinal study of Finnish adults, the researchers identified five personality types: the resilient, overcontrolled, undercontrolled, reserved, and ordinary groups (Kinnunen et al., 2012). Overall, resilient/protected individuals typically have low neuroticism and high levels of the other four dimensions. Overcontrolled/vulnerable individuals are exactly the opposite, with high neuroticism and low levels of the other dimensions. Undercontrolled individuals are characterized by low conscientiousness, slightly high neuroticism, and high openness and extraversion (Zhang et al., 2015). Reserved people usually have low openness. Moreover, all personality traits of moderate/ordinary individuals are at the middle levels (Kinnunen et al., 2012).

As a typical individual difference variable, the role of personality on sexual psychology and behavior has also been widely studied. Eysenck and Wilson (1976) suggested that extroverts are thrill seekers, while neurotic people do not enjoy sexual behavior as much because they are extremely tense. Moreover, extraversion is positively correlated with sexting, while agreeableness and emotional stability are negatively correlated (Delevi and Weisskirch, 2013). Openness is negatively correlated with sexual prejudice, for people who are higher in openness are more likely to accept social changes (Miller et al., 2012). Furthermore, personality types and personality disorders caused by the mixture of multiple personality traits are also related to psychosexual adjustment. Children of the undercontrolled type are more likely to have sex before the age of 16 (Atkins and Hart, 2008). Women with borderline personality disorder have been found to have chaotic relationships and more sexual partners in a short period of time (Thompson et al., 2019). However, previous studies on the influence of personality on psychosexual health have mostly focused on unhealthy sexuality and morbid personality and have not explored the normal groups. Therefore, we proposed the second hypothesis:

*H2*: Psychosexual adjustment differs across different personality types.

### The Moderating Effects of Personality

There is an interaction between personality traits and leisure satisfaction. Scholars generally hold that leisure has a profound and unique reflection on our personality (Munusturlar and Argan, 2016). Furthermore, the relationship between personality and leisure satisfaction has physiological basis. Eysenck and Wilson (1976) suggested that extroverts have high cortical inhibition and low baseline arousal, which causes them to seek external stimulation. Introverts, on the other hand, have a low arousal threshold and need less stimulation to maintain optimal levels. People with lower emotional stability are less playful and get less enjoyment from social activities (Lepp et al., 2015). Additionally, people of neuroticism may feel unfulfilled in leisure and be less cheerful than other personality types (Codish and Ravid, 2015). People of the traditional type of personality can obtain higher educational satisfaction in leisure than the investigative, enterprising, realistic, and artistic types. They score higher on social satisfaction than the enterprising type as well (Park, 2009).

We have found the relationship between leisure satisfaction and psychosexual adjustment as well as the relationship between personality and psychosexual adjustment. However, as far as we know, no one has investigated the relationship among these three variables. Under the theoretical framework of personal-environment fit models (Iso-Ahola and Weissinger, 1990), studies conducted have proved that the overcontrolled adolescent maladaptive behavior (such as criminal behavior) is a strategy to buffer leisure boredom (Spaeth et al., 2015). Moreover, it has been shown that there is an interaction between leisure satisfaction and personality on subjective well-being (Liu, 2014). Based on the research mentioned above, we proposed the third hypothesis:

*H3*: Leisure satisfaction and personality have an interactive effect on psychosexual adjustment.

**Figure 1:**
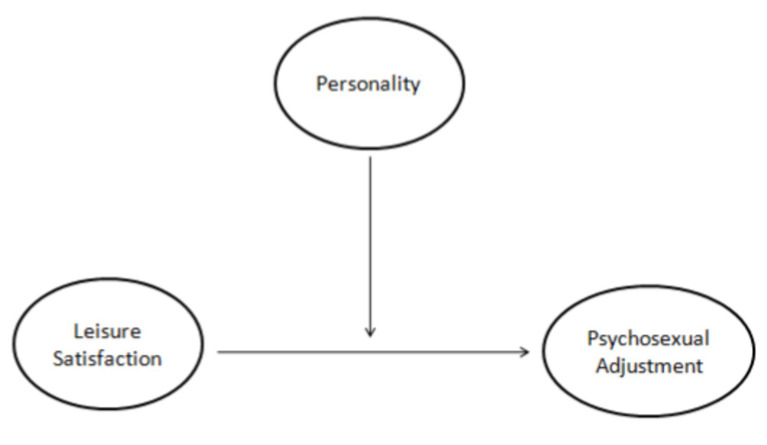
The moderation model used in this study.

In general, this study aims to explore the relationship between leisure satisfaction and psychosexual adjustment based on the compensation theory and the social learning theory. We also explored the differences in the psychosexual adjustment across different personality types. Furthermore, we examined the moderating effect of personality types between leisure satisfaction and psychosexual adjustment. We propose the following three hypotheses: H1: Leisure satisfaction is positively correlated with psychosexual adjustment. H2: Psychosexual adjustment differs across different personality types. H3: Leisure satisfaction and personality have an interactive effect on psychosexual adjustment. The theoretical model is shown in Figure 1.

## Materials and Methods

### Participants and Design

Participants in the study were undergraduate students from a university in China. The research team recruited participants through posters and online notifications. Our team informed them of the principles of confidentiality and voluntary. Participants would take part in the survey in designated classrooms at specified time, and would not get rewarded. In addition, the survey would be done anonymously and the results would be kept private. Eventually, a total of 1,161 students were recruited to answer the questionnaires. Data collecting was conducted from November 2021 to January 2022. One research group member and one school teacher were designated and trained as the experimenters. To start with, the research group member read the instructions. The participants were asked to complete the questionnaires in 25 min.

Invalid questionnaires (answering regularly or missing more than five questions) were removed after all the questionnaires were collected. Following the removal, 1,075 valid questionnaires were obtained. The rate of effective recovery was 92.6%. The average age of the participants was 18.7 (SD = 1.07). There were 511 males (47.5%) and 564 females (52.5%) participants. A total of 652 (60.7%) participants were rural residents, and 423 (39.3%) were urban residents.

### Measures

#### Leisure Satisfaction

The leisure satisfaction questionnaire for university students (Bingzheng et al., 2010) was used for the measurement of leisure satisfaction. It consists of three factors: relaxation satisfaction, contact satisfaction and transcendent satisfaction. Relaxation satisfaction refers to evaluation of the rest function of leisure. It contains six items such as “Leisure activities can help me relax.” Contact satisfaction refers to evaluation of communication function of leisure. It contains five items such as “My social interaction with others improves during leisure time.” Transcendent satisfaction refers to evaluation of self-development function of leisure. It contains six items such as “Leisure can improve my abilities.” The Cronbach’s alphas of each factor were 0.95, 0.92, and 0.93, respectively in this study. And the Cronbach’s alpha of the whole scale was 0.97 in this study. A 5-point Likert scale was adopted (1 represents very dissatisfied, 2 represents relatively dissatisfied, 3 represents neutral, 4 represents relatively satisfied, and 5 represents very satisfied). A higher score indicated a higher degree of leisure satisfaction.

#### Psychosexual Adjustment

The puberty psychosexual health scale (Yi and Yong, 2006) was used for the assessment of psychosexual adjustment. The scale was based on the previous studies (Dekker et al., 2017; Alley and Strassberg, 2020), which composed of three factors (sexual cognition, sexual values, and psychosexual adjustment). We used the psychosexual adjustment subscale, which contains three dimensions (self-adjustment, social adjustment, and sexual control). Specifically, self-adjustment refers to the adjustment to gender roles and changes in sexual characteristics. Social adjustment refers to the ability of expressing affection and satisfying sexual desires in ways that conform to sociocultural norms. And sexual control refers to the control over sexualized behavior. Self-adjustment involves five questions including “I am very satisfied with my gender.” Social adjustment consists of nine questions including “I express my affection in a similar way to my peers.” Sexual control contains six questions including “I cannot help but watch some pornographic publications or programs.” Multiple fit indices for psychosexual adjustment were evaluated in confirmatory factor analysis. The results showed that the *χ*^2^/*df* ratio was 7.64, CFI = 0.82, GFI = 0.89, and AGFI = 0.86 in this study. The Cronbach’s alpha of psychosexual adjustment in this study was 0.81. A 5-point Likert scale was adopted (1 represents strongly disagree, 2 represents relatively disagree, 3 represents neutral, 4 represents relatively agree, and 5 represents strongly agree). A higher score indicated a higher level of psychosexual adjustment.

#### Personality

The neuroticism extraversion openness five-factor inventory (NEO-FFI; Yao and Liang, 2010) was used for the assessment of personality. The NEO-FFI measures five personality traits: extraversion, openness, agreeableness, conscientiousness, and neuroticism. There are 12 items for each trait, with a total of 60 items. The extraversion subscale contains items including “I like being around people.” The openness subscale contains items like “I do not like fantasy.” The agreeableness subscale contains items including “Most of the people I know like me.” The conscientiousness subscale contains items like “I work tirelessly to reach my goals “. The neuroticism subscale contains items including “I have no worries.” The Cronbach’s alphas of the NEO-FFI in this study were 0.77 for extraversion, 0.67 for openness, 0.71 for agreeableness, 0.82 for conscientiousness, and 0.84 for neuroticism. A 5-point Likert scale was adopted in the questionnaire (1 represents strongly disagree, 2 represents relatively disagree, 3 represents neutral, 4 represents relatively agree, and 5 represents strongly agree). A higher score indicated a higher level of the trait.

### Data Analysis

Mplus 8.0 was used for the confirmatory factor analysis and classification of personality types using latent profile analysis. SPSS 23.0 was used for the descriptive statistics analysis and Pearson correlation analysis. The difference in psychosexual adjustment across different personality types was analyzed based on the hierarchical linear models in SPSS. The moderating effects of personality on leisure satisfaction (independent variable) and psychosexual adjustment (dependent variable) were tested based on the hierarchical multiple regression models in SPSS. And *p* < 0.05 was set as of statistical significance.

## Results

### Common Method Bias

The Harman single-factor test was used to test the method bias. The variance explained by the first factor (“I have passion in leisure activities” from leisure satisfaction questionnaire) was 20.14%, below the 40% threshold. The result of the principal component factor analysis without rotation showed that 19 factors had eigenvalues greater than 1. Therefore, common method bias did not affect the results of this study.

### Descriptive Statistics and Correlations

To test hypothesis 1, we used Pearson correlations. The means *(M)* and standard deviations (SD) of personality, leisure satisfaction and psychosexual adjustment are shown in Table 1. Table 1 also shows the correlation matrix of these variables. Among all five personality traits, extraversion, openness, agreeableness, and conscientiousness were positively correlated with leisure satisfaction and psychosexual adjustment (*p* < 0.01). Neuroticism was negatively correlated with leisure satisfaction and psychosexual adjustment (*p* < 0.01). Leisure satisfaction was positively correlated with psychosexual adjustment (*p* < 0.01).

**Table 1:** Descriptive statistics and correlations between personality, leisure satisfaction, and psychosexual adjustment (*r*, *n* = 1,075).

Variable	*M*	SD	1	2	3	4	5	6	7	8	9	10	11	12
1. Extraversion	3.24	0.53	–											
2. Openness	3.45	0.48	0.26^**^	–										
3. Agreeableness	3.87	0.47	0.33^**^	0.34^**^	–									
4. Conscientiousness	3.47	0.54	0.55^**^	0.39^**^	0.39^**^	–								
5. Neuroticism	2.80	0.62	−0.61^**^	−0.23^**^	−0.48^**^	−0.47^**^	–							
6. Relaxation satisfaction	4.07	0.64	0.36^**^	0.38^**^	0.31^**^	0.42^**^	−0.26^**^	–						
7. Contact satisfaction	3.90	0.66	0.51^**^	0.28^**^	0.31^**^	0.46^**^	−0.36^**^	0.71^**^	–					
8. Transcendent satisfaction	3.96	0.64	0.46^**^	0.37^**^	0.26^**^	0.48^**^	−0.30^**^	0.79^**^	0.80^**^	–				
9. Leisure satisfaction	3.98	0.60	0.48^**^	0.38^**^	0.32^**^	0.49^**^	−0.33^**^	0.91^**^	0.90^**^	0.94^**^	–			
10. Self-adjustment	3.94	0.63	0.45^**^	0.24^**^	0.30^**^	0.45^**^	−0.40^**^	0.36^**^	0.42^**^	0.40^**^	0.43^**^	–		
11. Social adjustment	3.71	0.54	0.32^**^	0.33^**^	0.32^**^	0.41^**^	−0.30^**^	0.38^**^	0.39^**^	0.40^**^	0.42^**^	0.51^**^	–	
12. Sexual control	3.42	0.74	0.11^**^	0.10^**^	0.32^**^	0.30^**^	−0.24^**^	0.12^**^	0.09^**^	0.09^**^	0.11^**^	0.07^*^	0.13^**^	–
13. Psychosexual adjustment	3.68	0.44	0.40^**^	0.32^**^	0.45^**^	0.54^**^	−0.43^**^	0.40^**^	0.41^**^	0.42^**^	0.45^**^	0.68^**^	0.81^**^	0.60^**^

* *p < 0.05*,

** *p < 0.01*.

*Leisure satisfaction is the sum of relaxation satisfaction, contact satisfaction, and transcend satisfaction. Psychosexual adjustment is the sum of self-adjustment, social adjustment and sexual control*.

### Latent Profile Analysis

To explore the potential patterns of personality traits, this study used latent profile analysis (LPA) with five personality traits as observation variables and one profile as the starting point. The results are shown in Table 2.

**Table 2:** Latent profile models (*n* = 1,075).

Number of profiles in model	AIC	BIC	ABIC	Entropy	LMR (*p*)
1	35052.23	35102.03	35070.27		
2	34020.55	34100.23	34049.41	0.77	<0.001
3	33758.82	33868.38	33798.50	0.80	<0.001
4	33624.39	33763.83	33674.90	0.83	<0.001
5	33562.63	33731.95	33623.96	0.74	0.145

*AIC, Akaike information criterion, BIC, Bayesian information criterion, ABIC, sample-size-adjusted BIC, and LMR (*p*), value of *p* for Lo–Mendell–Rubin*.

Table 2 contains the AIC, BIC, ABIC, entropy, and LMR statistics for each of the tested models. An optimal model fit is defined by lower AIC, BIC, and ABIC values (Finch, 2015). For all models, these three values decreased as the number of profiles increased from 1 to 5, providing support for the 5-profile solution. If the LMR value of model *K* reached the significance level (*p* < 0.05), it indicated that model *K* had a higher variance explanation rate than model *K*-1. According to the model fitting results in Table 2, the LMR values of the 2-profile model, 3-profile model and 4-profile model all reached the level of significance, showing that these three models were superior to the 5-profile solution. Furthermore, there was little difference in the index between the 4-profile model and the 5-profile model. Therefore, we preferred the 4-profile solution. Entropy represents classification accuracy, which generally has a high standard of 0.80 (Clark, 2010). Ultimately, the 4-profile model was chosen as the optimal latent profile analysis model.

Table 3 shows the *Z* scores of five personality traits for each personality profile. The overcontrolled group has higher neuroticism but lower scores in the other four dimensions, and Profile 1 matched these features. Profile 4 conformed to the definition of the resilient group, showing the lowest level of neuroticism and higher levels of the other four dimensions. All personality dimensions of Profile 2 and Profile 3 were almost at the average level. The only difference was that the scores of Profile 2 were slightly lower than those of Profile 3. Therefore, Profile 2 and Profile 3 were identified as typical personality types, with Profile 2 named the low-moderate group and Profile 3 named the high-moderate group.

**Table 3:** Statistical description of 4-profile model.

Profile	*n*	*%*	*Z* scores				
			Extraversion	Openness	Agreeableness	Conscientiousness	Neuroticism
1	50	4.7	−1.82	0.04	−0.94	−1.28	1.78
2	371	34.5	0.66	0.40	0.54	0.65	−0.69
3	608	56.6	−0.41	−0.36	−0.36	−0.44	0.43
4	46	4.3	2.12	1.41	1.48	1.95	−2.05

### Hierarchical Linear Models

**Figure 2:**
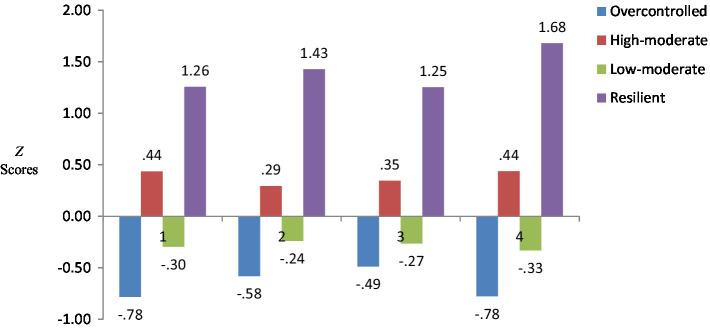
*Z* scores for each profile (*n* = 1,075). Note: 1, self-adjustment, 2, social adjustment, 3, sexual control, 4, psychosexual adjustment, psychosexual adjustment is the sum of self-adjustment, social adjustment, and sexual control.

For the test of hypothesis 2, bar charts were used to show the differences in the *Z* scores of college students with different personality types in psychosexual adjustment (see Figure 2). Furthermore, four types of personality were included in the hierarchical linear models. Profiles 1, 2, and 3 were compared as reference groups to test their differences in self-adjustment, social adjustment, sexual control, and psychosexual adjustment. The results are shown in Table 4. There was no significant difference between Profile 1 and Profile 3 in the sexual control dimension (*p* = 0.326). Except for that dimension, all personality types showed differences in psychosexual adjustment.

**Table 4:** Hierarchical linear models (*n* = 1,075).

Dependent variable	Profile	*t*	SE	*p*
**Self-adjustment**				
Profile1	Profile 2	−9.09	0.42	<0.001
	Profile 3	−3.72	0.41	<0.001
	Profile 4	−11.22	0.57	<0.001
Profile 2	Profile 3	10.03	0.18	<0.001
	Profile 4	−6.74	0.45	<0.001
Profile 3	Profile 4	−9.03	0.46	<0.001
**Social adjustment**				
Profile 1	Profile 2	−6.35	0.68	<0.001
	Profile 3	−2.55	0.66	0.011
	Profile 4	−10.76	0.92	<0.001
Profile 2	Profile 3	7.26	0.29	<0.001
	Profile 4	−8.57	0.71	<0.001
Profile 3	Profile 4	−10.38	0.70	<0.001
**Sexual control**				
Profile 1	Profile 2	−3.44	0.11	0.001
	Profile 3	−0.98	0.11	0.326
	Profile 4	−0.20	0.15	<0.001
Profile 2	Profile 3	4.90	0.05	<0.001
	Profile 4	−3.67	0.11	<0.001
Profile 3	Profile 4	−4.94	0.11	<0.001
**Psychosexual adjustment**				
Profile 1	Profile 2	−9.12	0.06	<0.001
	Profile 3	−3.45	0.06	0.001
	Profile 4	−13.44	0.08	<0.001
Profile 2	Profile 3	10.67	0.03	<0.001
	Profile 4	−9.53	0.06	<0.001
Profile 3	Profile 4	−11.86	0.06	<0.001

*Profile 1, overcontrolled group; Profile 2, high-moderate group; Profile 3, low-moderate group; Profile 4, resilient group*.

### Moderating Analysis

For the test of hypothesis 3, the hierarchical multiple regression models were used to investigate the moderating effects of personality on leisure satisfaction and psychosexual adjustment. Among the four personality types, we selected the overcontrolled group as the reference group. Three dummy codes were used for the high-moderate group, the low-moderate group, and the resilient group. To reduce the collinearity between variables, we standardized the independent variable (leisure satisfaction) first. After that, the interaction term was calculated between the standardized independent variable and the moderating variable (personality types). Leisure satisfaction was put into the first layer. Then, dummy codes of the moderating variable were added to the second layer. Finally, the interaction terms were added into the regression equation as the third level. The results show that the interaction of the leisure satisfaction and the dummy variable for the high-moderate group, the low-moderate group, and the resilient group was all statistically significant, with *p* < 0.001, *p* < 0.001, and *p* = 0.011, respectively, as shown in Table 5.

**Table 5:** Hierarchical multiple regression models with psychosexual adjustment as dependent variable.

Levels		*F*	*R^2^*	*β*	SE	*p*	*t*
1	Leisure satisfaction	332.32^***^	0.24	0.49	0.019	<0.001	18.28
2	Leisure satisfaction	108.23^***^	0.29	0.42	0.062	<0.001	4.88
	Resilient			0.23	0.053	<0.001	6.05
	High-moderate			0.07	0.044	0.167	1.38
	Low-moderate			−0.06	0.040	0.395	−0.85
3	Leisure satisfaction	83.49^***^	0.35	0.19	0.073	0.057	1.91
	Resilient			0.11	0.104	0.153	1.43
	High-moderate			0.09	0.049	0.131	1.52
	Low-moderate			0.07	0.046	0.348	0.94
	*Z* leisure satisfaction ×						
	Resilient			0.32	0.154	<0.001	4.29
	*Z* leisure satisfaction ×						
	High-moderate			0.43	0.064	<0.001	6.09
	*Z* leisure satisfaction ×						
	Low-moderate			0.18	0.062	0.011	2.55

*** *p < 0.001*.

**Figure 3:**
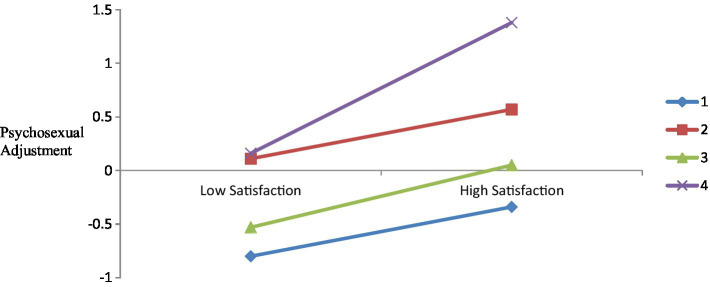
A simple slope plot of the moderating effect (*n* = 1,075). Note: 1, overcontrolled group, 2, high-moderate group, 3, low-moderate group, 4 = resilient group.

To further explore the moderating effects of personality, we divided participants into two groups: a low leisure satisfaction group (scored one standard deviation below the average) and a high leisure satisfaction group (scored one standard deviation above the average). Figure 3 illustrates the moderating effects of personality. For the accurate demonstration of the moderating effects of each personality type, a linear regression model test was conducted for each group and the path coefficients were 0.24 for the overcontrolled group (*t* = 1.74, *p* = 0.089), 0.22 for the high-moderate group (*t* = 4.28, *p* < 0.001), 0.30 for the low-moderate group (*t* = 7.75, *p* < 0.001), and 0.47 for the resilient group (*t* = 3.49, *p* = 0.001).

## Discussion

Overall, our study verified our three hypotheses, providing certain basis for the improvement of college students in psychosexual adjustment. First, there was a positive relationship between leisure satisfaction and psychosexual adjustment. Moreover, college students with different personality types have different performance in psychosexual adjustment in our study. In addition, personality and leisure satisfaction have an interactive effect on psychosexual adjustment. In general, good leisure experiences may improve individual psychosexual adjustment of all personality types.

### Leisure Satisfaction and Psychosexual Adjustment

As expected, the results confirmed a link between leisure satisfaction and psychosexual adjustment. Consistent with previous research (Zerengok et al., 2018), when college students actively participated in leisure activities, they showed progress in social adjustment and reported psychological and physical benefits. This result can be interpreted based on social learning theory. People learn communication skills and norms, and learn to understand themselves and others appropriately through entertainment on informal occasions. Individuals could derive pleasure and social benefits from interpersonal interactions in leisure activities (Chao, 2013). In other words, positive leisure can enable people to achieve psychosexual socialization (Shin and You, 2013). Apart from this, leisure is regarded as a potential resource for people in dealing with some forms of stigma such as sexual orientation (Mock et al., 2013). Leisure could provide sexual minorities with a sense of belonging which brings benefits to their psychosexual selfhood, but this requires favorable social and relaxing conditions (Outley and McKenzie, 2007; Jones and McCarthy, 2010). As previous research has found, engaging in meaningful social leisure activities can positively impact current and future mental health (Timonen et al., 2021).

Based on the compensation theory, positive experience in leisure seems to be a powerful source of self-control efficacy (Stebbins, 2006). Due to the lack of leisure resources and opportunities, bored young people may turn to risky activities (Sharp et al., 2011). Leisure interventions that can change the leisure cognition of teenagers and help improve their leisure satisfaction, such as HealthWise (Motamedi et al., 2020), have been shown having the potential to reduce the likelihood of early sexual activity in boys.

Among all the relationships between leisure satisfaction and psychosexual adjustment, the relationship between contact satisfaction and self-adjustment was the strongest. One explanation is that self-esteem can be gained from social support (Erango and Ayka, 2015). When people get along well with others and receive positive feedback about themselves from others, they can improve their self-acceptance concerning physiology and psychology. This finding was also expressed by Cooley’s conception of the “looking-glass self” (Cooley, 1902), in which the actor constantly builds his self-image based on the reactions of others to his performances. Similarly, social interaction has explained more than half of the association between green space use and self-satisfaction (Dadvand et al., 2019).

### Personality and Psychosexual Adjustment

We further explored the difference in psychosexual adjustment across different personality types. In the first place, we used the same five-factor model to identify different personality types as many human-centered studies do. Our results were consistent with the findings of other researchers but somewhat different. We found that the vast majority of Chinese college students in our research could be classified as the moderate group (there were differences between the high- and low-moderate groups), a small number of students (approximately 4.7%) could be classified as the resilient group and approximately 4.3% could be classified as the overcontrolled group. Unlike a previous study (Alessandri et al., 2014), we did not actually identify an undercontrolled group. This result may, in part, be explained by the stricter control of Chinese parents over teenagers and the corresponding more reserved qualities teenagers have (Ho, 1994; Wang et al., 2018).

The hierarchical linear models seem to indicate that the overcontrolled group was at a disadvantage in all dimensions of psychosexual adjustment compared with other groups. Only in the sex control dimension was there no significant difference between the overcontrolled group and the low-moderate group. The overcontrolled group scored relatively low in the dimension of extraversion and relatively high in neuroticism, showing inflexibility in cognition and behavior and high attention to detail processing (Gilbert et al., 2020). Besides, the overcontrolled group is usually associated with a higher likelihood of internalizing problems (Asendorpf et al., 2001). This situation is mainly due to the fact that overcontrolled individuals may have more difficulty participating in social activities or engaging fully in intimate relationships (Denissen et al., 2008). Nevertheless, this situation also entails that such individuals have a higher degree of control than undercontrolled people (Block and Block, 1980). This fact may explain why the overcontrolled group did not differ from the low-moderate group in sexual control.

The resilient group, the opposite of the overcontrolled group, showed an advantage across all dimensions. Adolescence is a time when young people can sort out their sexual identities and sexual orientation (Katz-Wise, 2015). Meanwhile, in this phase the youth might worry about their sexual attractiveness. Individuals in the resilient group excel at this stage because they are able to adapt to a variety of environmental needs (Asendorpf, 2006). Specifically, higher levels of resilience are associated with higher levels of self-acceptance and self-control (Baeva et al., 2016), both of which contribute to the development of psychosexual adjustment (Woodford et al., 2014; Magnusson et al., 2019).

Comparing Profiles 2 and 3, both of which were considered moderate, we found that the former profile performed better in psychosexual adjustment. But overall, they scored in the middle in psychosexual adjustment. Consistent with previous findings (Rettew et al., 2008), individuals with moderate characteristics belong to a stable class. They do not pursue stimulating experience too excessively and have a certain degree of self-protection. In addition, more agreeable and more open individuals may have greater psychological and physiological adjustment (Boyce and Wood, 2011; Ó Súilleabháin et al., 2018).

### The Moderating Effects of Personality

Considering people with different personality types process information in different ways, we analyzed the interaction between leisure satisfaction and personality to understand how individuals apply leisure experience. The moderating effects of all personality types were positive, and the moderating effect of the resilient group was the most prominent. These results confirmed the applicability of personal-environment fit models to adolescent psychosexual adjustment. As a risk-protective factor, resilience can promote positive development during times of adversity (Luthar et al., 2000). In high-risk family settings, resilient youth are 3.5 times more likely to be free of health problems throughout their lifetime than their peers with low resilience (Hopkins et al., 2015). The moderating effects of the remaining personality types were also positive, but not as good as that of the resilience group. Moreover, it is worth emphasizing that leisure satisfaction seems to make a major contribution to the existence of a moderating effect. This further suggests that adolescents of different types could benefit from well-experienced leisure (Bradley and Inglis, 2012).

## Conclusion

Our aim was to investigate the relationship among leisure satisfaction, personality, and psychosexual adjustment. The following conclusions were drawn: (1) within the framework of the compensation theory and social learning theory, there is a positive correlation between leisure satisfaction and psychosexual adjustment; (2) psychosexual adjustment differs across different personality types. The resilient group excelled at all dimensions, while the overcontrolled group scored lowest at all dimensions. The high-moderate group and the low-moderate group scored moderately, and the high-moderate group scored higher than the low-moderate group; (3) within the framework of the personal-environment fit models, the moderating effect of personality types has been verified, and the resilient group performed best.

## Limitations

First of all, leisure satisfaction has possible effects on the psychosexual adjustment of adolescents, psychosexual adjustment may also in turn affect leisure satisfaction. For example, to a certain extent, more decent and more socially compliant interactions with others mean higher levels of contact satisfaction in leisure. Accordingly, we may need longitudinal studies to explore deeply the relationship between these two. In addition, there were certain geographical limitations concerning our research participants. Geographical differences could lead to huge differences in economic conditions, lifestyles, and ultimately differences in characteristics. Furthermore, we did not find the undercontrolled group and the reserved group in our study. Instead, we found that the moderate group could be divided into two types, which is different from previous studies. This is also one of the limitations of our research. For an accurate study of personality types, the sampling scope of participants may be expanded. Finally, gender is also a point of interest for our future research, for personality and gender may have mixed effects on psychosexual adjustment. If this variable had been added, our research could be more enriched.

## Data Availability Statement

The original contributions presented in the study are included in the article/supplementary materials, further inquiries can be directed to the corresponding author.

## Ethics Statement

The studies involving human participants were reviewed and approved by Research Ethics Committee of Qingdao University. Written informed consent for participation was not required for this study in accordance with the national legislation and the institutional requirements.

## Author Contributions

XX: conceived and designed the survey. XW: performed the survey. YW and XW: analyzed the data. YW: contributed materials/analysis tools. XX and XW: wrote the paper and literature research. All authors contributed to the article and approved the submitted version.

## Funding

This work was supported by the National Social Science Fund of China (No.19BKS087) awarded to XX.

## Conflict of Interest

The authors declare that the research was conducted in the absence of any commercial or financial relationships that could be construed as a potential conflict of interest.

## Publisher’s Note

All claims expressed in this article are solely those of the authors and do not necessarily represent those of their affiliated organizations, or those of the publisher, the editors and the reviewers. Any product that may be evaluated in this article, or claim that may be made by its manufacturer, is not guaranteed or endorsed by the publisher.
